# Thanks to reviewers

**DOI:** 10.1007/s40037-012-0034-z

**Published:** 2012-12-01

The Editorial Board greatly acknowledges the advice of the reviewers. They made an important contribution to the quality of the journal.

Anthony Artino, Sanneke Bolhuis, Joseph Donald Boudreau, Johan Bredéé, Jamiu Busari, Patrick Cras, Menno de Bree, Esther de Groot, Peter de Jong, Benedicte De Winter, Joke Denekens, Diana Dolmans, Jos Draaisma, Michiel Eijkman, Petri Embregts, Frans Grosfeld, Harianne Hegge, Frank Krings, Jan Kuks, Rashmi Kusurkar, Mario Maas, Willemina Molenaar, Arko Oderwald, Karlijn Overeem, Martien Quaak, Roy Remmen, Jan-Joost Rethans, George Richard, Clemens Rommers, Judith Rosmalen, Johanna Schönrock-Adema, Robert Schoevers, Philippe Schucht, Lambert Schuwirth, Veronica Selleger, Arjun Singh, Renée Stalmeijer, Paul Stuyt, Edith ter Braak, Rene Tio, Nynke van Dijk, Paul Van Royen, Tineke Westerveld, David Whitford, Indah Widyahening, Margreet Wieringa-de Waard and Raniai Zaini.

